# The evolution of electrocardiographic abnormalities in the elderly with Chagas disease during 14 years of follow-up: The Bambui Cohort Study of Aging

**DOI:** 10.1371/journal.pntd.0011419

**Published:** 2023-06-07

**Authors:** Bruno Oliveira Figueiredo Brito, Emilly Malveira Lima, Elsayed Z. Soliman, Eduardo Fernandes Silva, Maria Fernanda Lima-Costa, Antonio Luiz Pinho Ribeiro

**Affiliations:** 1 Faculdade de Medicina, Universidade Federal de Minas Gerais, Belo Horizonte, Brazil; 2 Serviço de Cardiologia e Cirurgia Cardiovascular, Hospital das Clínicas, Universidade Federal de Minas Gerais, Belo Horizonte, Brazil; 3 Telehealth Center, Hospital das Clínicas, Universidade Federal de Minas Gerais, Belo Horizonte, Brazil; 4 Department of Epidemiology and Prevention, Wake Forest University School of Medicine, Winston-Salem, North Carolina, United States of America; 5 Fundação Oswaldo Cruz, Minas Gerais, Brazil; Federal University of Ceará, Fortaleza, Brazil, BRAZIL

## Abstract

**Background:**

The natural history of Chagas disease (ChD) in older ages is largely unknown, and it is a matter of controversy if the disease continues to progress in the elderly.

**Objective:**

To investigate the evolution of electrocardiographic abnormalities in *T*. *cruzi* chronically infected community-dwelling elderly compared to non-infected (NChD) subjects and how it affects this population’s survival in a follow-up of 14 years.

**Methods and results:**

A 12-lead ECG of each individual of the Bambui Cohort Study of Aging was obtained in 1997, 2002, and 2008, and the abnormalities were classified using the Minnesota Code. The influence of ChD on the ECG evolution was assessed by semi-competing risks considering a new ECG abnormality as the primary event and death as the terminal event. A Cox regression model to evaluate the population survival was conducted at a landmark point of 5.5 years. The individuals of both groups were compared according to the following categories: Normal, Maintained, New, and More by the development of ECG major abnormalities between 1997 and 2002. Among the participants, the ChD group had 557 individuals (median age: 68 years) and NChD group had 905 individuals (median age: 67 years). ChD was associated with a higher risk of development of a new ECG abnormality [HR: 2.89 (95% CI 2.28–3.67)]. The development of a new major ECG abnormality increases the risk of death ChD patients compared to those that maintain a normal ECG [HR: 1.93 (95% CI 1.02–3.65)].

**Conclusion:**

ChD is still associated with a higher risk of progression to cardiomyopathy in the elderly. The occurrence of a new major ECG abnormality in ChD patients predicts a higher risk of death.

## Author summary

Chagas disease (ChD) is caused by the protozoan parasite *Trypanosoma cruzi* and continues to be a health problem despite the control of its transmission. ChD is a heterogeneous condition with a wide variation in its clinical course and prognosis. The majority (60%–70%) of infected individuals remain asymptomatic throughout life. Chagas cardiomyopathy (ChCM) encompasses all cases of ChD with cardiac involvement. It is defined by the presence of at least a typical electrocardiographic abnormality in patients with positive serological tests against *T*. *cruzi*. There is very little reliable information about the natural history of ChD individuals in old age, and most community-based longitudinal studies have been conducted on young adults. The findings of this study suggest that the *T*. *cruzi* infection provides continuous damage to the myocardium and that it is an independent risk of the occurrence of new ECG abnormalities despite the other factors that could cause cardiac lesions and promote ECG abnormalities associated with aging. The newly developed cardiomyopathy in the elderly is as ominous as the cardiomyopathy established at earlier ages.

## Introduction

Chagas disease (ChD) is a neglected tropical disease caused by a protozoon, *Trypanosoma cruzi*, and affects nearly 6 million persons worldwide [1]. Due to migratory movements, ChD is now a problem also in non-endemic, high-income countries. The infection by the *T cruzi* in endemic areas occurs early in life, and almost all cases evolve into an indeterminate chronic form, defined by the positive serological test for *T*. *cruzi* and the absence of symptoms, signs, electrocardiographic and radiologic abnormalities suggestive of cardiac or digestive disease [1,2]. Patients with an indeterminate chronic form will progress to clinically manifest cardiomyopathy, at a rate of near 2% per year [3,4]. Chagas cardiomyopathy (ChCM) encompasses all cases of Chagas disease with cardiac involvement, defined by the presence of at least a typical electrocardiographic abnormality in patients with positive serological tests against *T cruzi* [1]. The enlargement of all 4 cardiac chambers without pulmonary congestion seen in the chest x-ray suggests Chagas cardiomyopathy. Cardiomegaly (a cardiac-to-thoracic ratio >50%) is an important predictor of mortality [1].

Since the initial descriptions of ChCM, the electrocardiogram (ECG) has played a key role in patient evaluation [5,6]. The ECG is a well-known method for diagnosing ChCM and defining prognosis [7]. Moreover, it is a low-cost and widely available exam, even in remote areas. During the course of the disease, the ECG shows progressive abnormalities that indicate worsening myocardial damage [5,8]. Most of the previous studies considered the ECG abnormalities in patients with Chagas disease as predictors of adverse outcomes [7], and few analyzed their progression in the course of the disease.

There is very little reliable information about the natural history of ChD individuals in old age, and most community-based longitudinal studies have been conducted on young adults [4]. Elderly individuals with Chagas disease were expected to have a more benign course of the disease and not develop new ECG abnormalities [5]. It was suggested that ECG abnormalities could be caused both by ChCM and common conditions of the elderly, such as ischemic and hypertensive heart disease [9].

Considering that there is an increasing number of elderly patients with Chagas disease due to a cohort effect, assessing its behavior in this population is of utmost importance. In the present study, baseline data and 14 years of follow-up of the Bambui Cohort Study of Aging in Brazil were used to investigate the evolution of electrocardiographic abnormalities in *T*. *cruzi* chronically infected compared to non-infected elderly and how it affects this population survival.

## Methods

### Ethics statement

This research was approved by the Ethics Committee of the Oswaldo Cruz Foundation—Minas Gerais, logged under protocol number of the Plataforma Brasil (CAAE: 34649814.3.0000.5091) on November 4^th^ of 1996. All participants provided written informed consent. Data are already available in the following public repository: https://elsi.cpqrr.fiocruz.br/data-access.

### Study population

The Bambui cohort study of aging is a population-based cohort study conducted in Bambui city (15,000 inhabitants), situated in Southeast Brazil. Bambui is one of the oldest known endemic areas for ChD. Despite successfully interrupting the transmission of T. cruzi infection by 1970, the disease has remained highly prevalent in the elderly due to a cohort effect [10,11]. Procedures used in the cohort study were described in detail elsewhere [10,11]. Briefly, the baseline cohort population comprised all residents aged ≥60 on January 1, 1997, who were identified by a complete census conducted in the town [10,11]. Baseline data collection was performed from February to May 1997, comprising standardized interviews, blood and clinical tests, and an electrocardiogram. Cohort members underwent annual follow-up examinations in arranged clinic visits. Participants signed informed consent and authorized death certificate verification.

### Mortality data source

**Fig 1:**
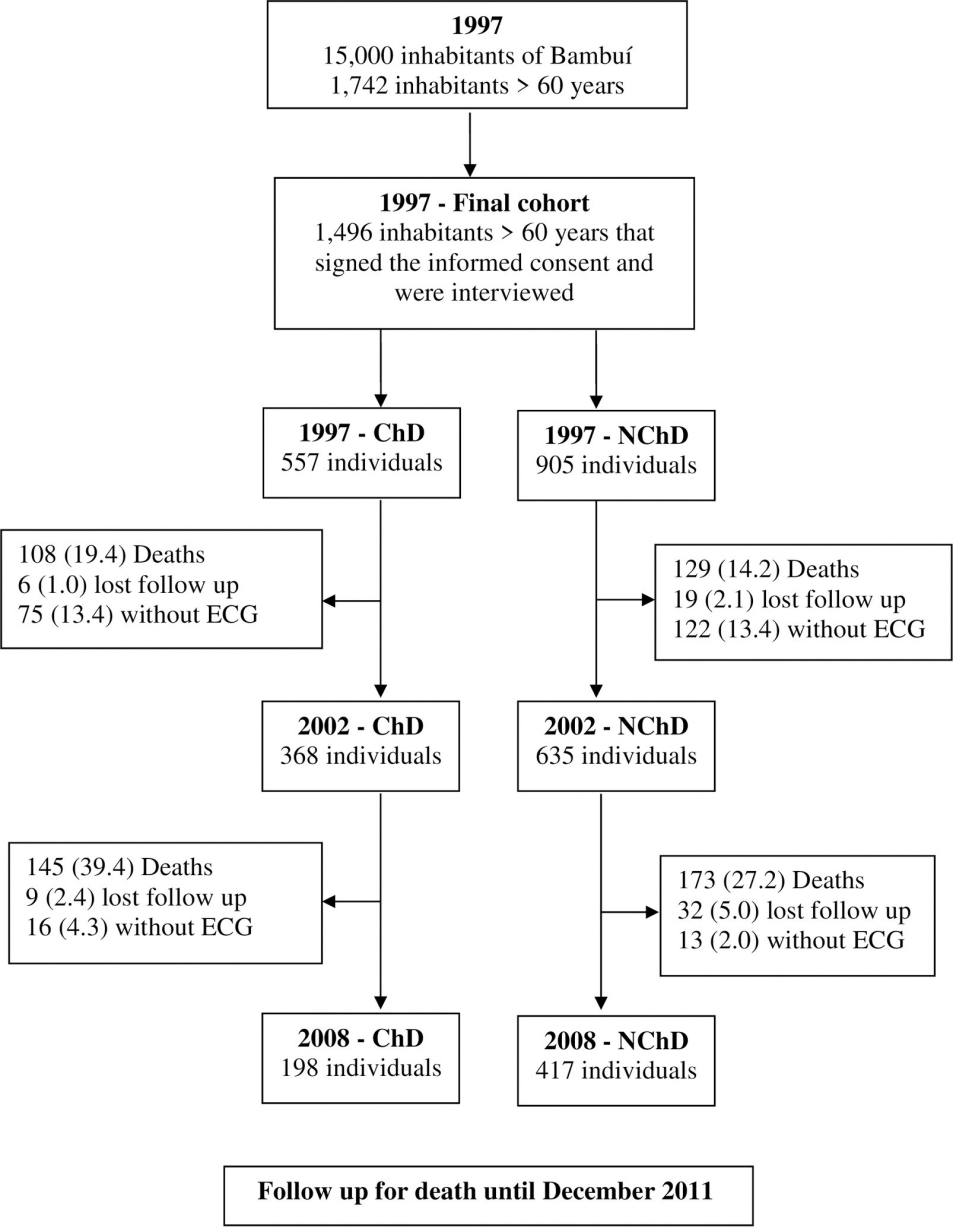
Flow chart describing the design of the study. Note: The values in parenthesis correspond to the percentages.

Deaths from any cause occurring from the beginning of the study enrollment in 1997 until December 31, 2011, were included in this analysis. They were reported by next of kin during the annual follow-up interview and ascertained through the Brazilian System of Information on Mortality, with the permission of the Ministry of Health. Death certificates were obtained for 98.9% of participants who died. Fig 1 describes the design of this study.

### Electrocardiogram

A digitally recorded 12-lead ECG (Hewlett Packard MI700A) was obtained at rest using standardized procedures at the baseline examination in 1997 and repeated in 2002 and 2008. ECGs were analyzed by experienced cardiologists at the ECG Reading Center (EPICARE, Wake Forest University) and visually classified by the Minnesota Code (MC) criteria [12]. QT interval was corrected to heart rate and reported as QT index (QTi), calculated as QTi = (QT/656)X (HR+100) [13]. In this study, major and minor ECG abnormalities were defined as set out in Prineas et al [14], modified to include ECG abnormalities typical of Chagas cardiomyopathy with prognostic significance, as frequent supraventricular or ventricular premature beats [15]. Major ECG abnormalities included old myocardial infarction (MI) (major Q-wave abnormalities [MC 1.1.x or 1.2.x]) or possible MI (minor Q-waves abnormalities with ST segment or T-wave abnormalities [1.3.x and 4.1.x, 4.2, 5.1, or 5.2]), complete intraventricular blocks (7.1, 7.2, 7.4, or 7.8), frequent supraventricular or ventricular premature beats (MC 8.1.x, except 8.1.4), major isolated ST segment or T-wave abnormalities (MC 4.1.x, 4.2, 5.1 or 5.2), atrial fibrillation or flutter or supraventricular tachycardia (MC 8.3.x. or 8.4.2), other major arrhythmias (MC 8.2.x, except 8.2.1), major atrioventricular conduction abnormalities or pacemaker use (MC 6.1, 6.2.x, 6.4, 6.8, 8.6.1 or 8.6.2), major QT prolongation (>115%) and left ventricular hypertrophy (LVH) (MC 3.1 together with [4.1.x, 4.2, 5.1, or 5.2]).

### *T*. *cruzi* infection status and B-type natriuretic peptide

Diagnosis of chronic ChD relies on serologic methods. Infection with *T*. *cruzi* was defined by seropositivity in 3 examinations, a hemagglutination assay (Bio-Merieux, France) and 2 enzyme-linked immunoabsorbent assays (Abbott and Viener). B-type natriuretic peptide (BNP) was measured using a microparticle-based immunoassay (MEIA/AxSYM; Abbott).

### Statistical analysis

Statistical analyses were conducted using SPSS 18.0 and R (3.6.1) Software. A descriptive analysis of the data with frequencies, means, medians of electrocardiographic variables, and major abnormalities was obtained. The Kolmogorov-Smirnov test was performed to evaluate normality. The variables with normal distribution were described as means and standard deviation, while those with asymmetric distribution were expressed as medians and the interquartile range. The Mann-Whitney test was applied to compare the medians. To compare categorical variables, the chi-squared test was used. A 2-tailed P value < 0.05 was considered statistically significant. The incidence rate was calculated by dividing the number of events within that time interval by the number of person-years, corresponding to the sum of times lived (in years) by each component at risk at baseline. The confidence interval was obtained by Bootstrap, with 10000 repetitions at each step, a sample of the same size is randomly selected with replacement of the original, and the rate is calculated. The 95% confidence interval is quantiles of order 2.5% and 97.5%.

Considering that many individuals could die before the occurrence of new electrocardiographic abnormalities, the ECG evolution was assessed by a semi-competing risks method. We conducted the semi-competitive risk analysis considering death and the occurrence of a new major abnormality in the ECG as the outcomes: a new ECG abnormality was the primary event, and death was the terminal event. The group with ChD was compared with the group Without ChD (NChD). ChD was the exposure variable, and the covariates were risk factors for cardiovascular disease—age, male sex, current smoking, body mass index (BMI), serum creatinine ≥ 1,5mg/dL, diabetes mellitus, systolic blood pressure (each 10mmHg). The analyses were based on three models. First, we estimated the crude association between chronic ChD and the occurrence of new major ECG abnormalities and death (model 1); adjusted for age and gender (model 2); and adjusted for age, gender, smoking, diabetes, creatinine ≥ 1.5, systolic pressure (each 10mmHg) and body mass index (model 3).

A secondary analysis was conducted to assess the effect of the occurrence of a new major ECG abnormality on mortality. A new variable that contains the information about ECG progression was defined in four categories: (1) Normal: without major abnormalities in the visits of 1997 and 2002; (2) Maintained: the number of major abnormalities in 2002 was equal to the visit of 1997, (3) New: individuals without major abnormalities in the visit of 1997 that developed any abnormality in the visit of 2002, and (4) More: individuals who already had abnormalities at the visit of 1997 and developed more abnormalities at the visit of 2002. Some individuals in the groups with ChD (58 individuals) and in NChD (68 individuals) have reduced number of abnormalities in 2002 compared to the number of abnormalities in 1997. Those individuals were not included in the analysis. The ChD and NChD groups were compared separately, considering these categories. A Cox proportional-hazards regression model was performed considering only those individuals who were alive and maintained the follow-up in a landmark point of 65 months until the end of the follow-up. This landmark was chosen because, at this point, the electrocardiograms of the second wave were available to compare with the ones of the first wave; from this landmark, the influence of a new ECG abnormality on mortality could be determined. The population analyzed was comprised of 879 individuals. The landmark analysis was conducted, considering death as the outcome. The exposure variables were the categories “Normal”, “Maintained”, “New”, and “More”, defined above. The covariates for this analysis were risk factors for cardiovascular disease—age, male sex, current smoking, BMI, serum creatinine ≥ 1,5 mg/dL, diabetes mellitus, and systolic blood pressure (each 10mmHg). This test was performed separately for ChD and NChD groups. The groups Normal and Maintained were used as references in this analysis.

## Results

### Baseline characteristics

Among the 1,462 participants in this study, 557 had ChD, and 905 were noninfected (NChD). The median age was 68 years for patients with ChD and 67 years for NChD (p = 0.14). Women predominated in both groups with a higher proportion in NChD (p<0.001). ChD group had a higher median value of BNP. Baseline characteristics of the study participants according to the serological status group are shown in Table 1.

**Table 1:** Baseline characteristics of the study participants according to the serological status group.

Characteristics	ChD (557)	NChD (905)	p
**Age (years)**	68 (64–74)	67 (63–73)	0.14
**Male gender**	32.5	43.3	< 0.001
**Body mass index**	24.38 ± 5.05	25.59 ± 4.83	< 0.001
**Creatinine**	0.85 (0.75–0.97)	0.85 (0.74–0.99)	0.98
**Systolic blood pressure, mmHg**	133 (119–149)	136 (124–151)	0.02
**BNP, pg/ml**	119.5 (63.0–206.5)	64.0 (35.0–112.0)	< 0.001
**Current smoker**	17.6	18.3	0.72
**Diabetes on treatment**	57 (10.3%)	152 (16.8%)	0.001
**Use of Anti-hypertensives**	285 (51.2%)	457 (50.5%)	0.80
**Use of Beta-blockers**	25 (4.5%)	96 (10.6%)	< 0.001
**Use of Amiodarone**	56 (10.1%)	20 (2.2%)	< 0.001
**Use of Digoxin**	124 (22.3%)	100 (11.0%)	< 0.001

### ECG Abnormalities in follow up

**Fig 2:**
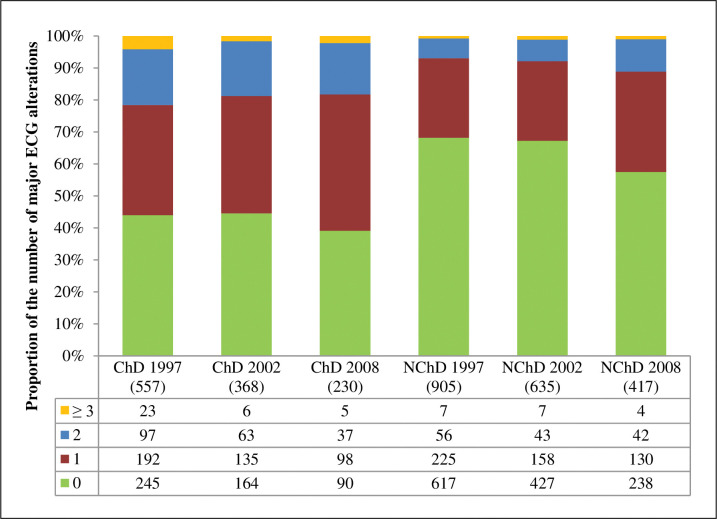
Number of major ECG alterations of the study participants according to the serological status group and the year.

The Fig 2 shows that major ECG abnormalities were more frequently found in ChD participants during the follow-up period. Individuals may have more than one abnormality in the ECG. In the Fig 2, they were categorized according to the number of abnormalities in their ECG. There is a reduction in the proportion of normal ECG and a rise in the proportion and the number of major abnormalities in both groups. Table 2 shows the prevalence of major ECG abnormalities in each group with the highest proportion of right bundle branch block (RBBB) and RBBB plus anterior fascicular block (AFB), major isolated ST-T abnormalities, and frequent supraventricular and ventricular premature beats during all the periods of follow-up in the group with ChD.

**Table 2:** Frequency of major ECG abnormalities according to the serological status group and year of examination.

Electrocardiographic Abnormalities	1997		2002		2008	
	**ChD (557)**	**NChD (905)**	**ChD (368)**	**NChD (635)**	**ChD (198)**	**NChD (417)**
**Major Q-wave abnormalities**	24 (4.3)	16 (1.7)	13 (3.5)	21 (3.3)	13 (6.5)	27 (6.4)
**Minor Q-wave plus ST-T abnormalities**	9 (1.6)	13 (1.4)	1 (0.2)	22 (3.4)	5 (2.5)	15 (3.6)
**Major isolated ST-T abnormalities**	63 (11.3)	118 (13.0)	33 (8.9)	54 (8.5)	23 (11.6)	49 (11.7)
**Left Bundle Branch Block**	18 (3.2)	18 (2.0)	20 (5.4)	26 (4.1)	14 (7.0)	20 (4.8)
**Intraventricular block**	3 (0.5)	1 (0.1)	7 (1.9)	5 (0.8)	4 (2.0)	8 (1.9)
**Right Bundle Branch Block**	78 (14.0)	22 (2.4)	13 (3.5)	10 (1.5)	10 (5.0)	6 (1.4)
**RBBB + AFB**	51 (9.1)	8 (0.8)	68 (18.5)	14 (2.2)	39 (19.7)	17 (4.0)
**Atrial fibrillation**	32 (5.7)	17 (1.8)	27 (7.3)	14 (2.2)	14 (7.0)	17 (4.0)
**Atrial flutter**	2 (0.3)	0 (0.0)	1 (0.2)	2 (0.3)	1 (0.5)	1 (0.2)
**Pacemaker**	0 (0.0)	6 (0.6)	11 (3.0)	3 (0.4)	11 (5.5)	2 (0.5)
**2nd AV block**	0 (0.0)	0 (0.0)	0 (0.0)	0 (0.0)	0 (0.0)	0 (0.0)
**3rd AV block**	3 (0.5)	1 (0.0)	1 (0.2)	0 (0.0)	0 (0.0)	0 (0.0)
**Supraventricular tachycardia**	7 (1.2)	3 (0.3)	2 (0.5)	2 (0.3)	1 (0.5)	0 (0.0)
**Frequent supraventricular or ventricular premature beats**	116 (20.8)	87 (9.6)	62 (16.8)	63 (9.9)	38 (19.2)	37 (8.8)
**Major QT prolongation (>115%)**	33 (5.9)	19 (2.1)	13 (3.5)	13 (2.0)	13 (6.5)	18 (4.3)
**Left ventricular hypertrophy**	11 (1.9)	37 (4.1)	5 (1.3)	25 (3.9)	2 (1.0)	16 (3.8)
**Major ECG abnormalities**	312 (56.0)	288 (31.8)	204 (55.4)	208 (32.7)	140 (70.7)	179 (42.9)

Note: The parenthesis values correspond to the percentages.

Table 3 shows the incidence of a new major ECG abnormality by serological status during the follow-up in the group with no major abnormalities in the baseline and the group with any major abnormality in the baseline from 1997 to 2008. In the group with ChD with No abnormalities, there were 57 new-onset ECG abnormalities from 1997 to 2008, resulting in an incidence of 24.8 (95% CI 19.5–30.1) events /1000 person-years and, in the NChD group, 92 new-onset ECG abnormalities resulting in an incidence of 15.5 (95% CI 12.7–18.5) events /1000 person-years. The absolute incidence difference associated with ChD in the overall period comprising 1997 to 2008 was 9.25 (95% CI 3.2–15.2). The absolute incidence difference associated with ChD was not significant for the individuals with any ECG abnormality in the baseline. The highest incidence of major ECG abnormalities in the ChD is noticeable, particularly of the following: frequent supraventricular or ventricular premature beats, RBBB + AFB, major isolated ST-T abnormalities, and atrial fibrillation (Table 4).

**Table 3:** Incidence of a new major ECG abnormality according to the serological status during the follow-up in the group with no major abnormalities in the baseline and in the group with any major abnormality in the baseline 1997–2008.

	Number at risk	Follow-up time (person-years)	Number of events	Incidence events/1000 person-years (95% CI)	Absolute incidence difference (95% CI)
**No major ECG abnormality in the baseline**					
** NChD**	617	5789	92	15.5 (12.7–18.5)	Reference
** ChD**	245	2298	57	24.8 (19.5–30.1)	9.25 (3.2–15.2)
**Any major ECG abnormality in the baseline**					
** NChD**	288	2315	28	12.1 (1.3–16.4)	Reference
** ChD**	312	2397	24	10.0 (0.0–13.8)	-2.1 (-13.8–8.1)

**Table 4:** Incidence of new major ECG abnormalities by serological status, per 1,000 person-year, during the follow-up from 1997 to 2008.

Electrocardiographic Abnormalities	Number of events		Incidence events (95% CI)	
	**ChD**	**Non ChD**	**ChD**	**Non ChD**
**Major Q-wave abnormalities**	11	16	4.4 (2.0–7.2)	5.3 (3.3–7.5)
**Minor Q-wave plus ST-T abnormalities**	5	8	2.0 (0.4–3.9)	3.0 (1.5–4.8)
**Major isolated ST-T abnormalities**	14	27	6.0 (3.0–9.0)	7.0 (4.6–9.5)
**Left Bundle Branch Block**	10	15	4.0 (1.6–6.5)	2.9 (1.3–4.4)
**Right Bundle Branch Block**	5	4	2.2 (0.4–4.4)	0.9 (0.2–1.8)
**Intraventricular block**	4	5	1.6 (0.4–3.1)	1.7 (0.6–3.0)
**RBBB + AFB**	25	11	10.6 (6.8–14.5)	3.7 (2.0–5.5)
**Atrial fibrillation**	12	8	4.8 (2.4–7.5)	2.8 (1.5–4.4)
**Atrial flutter**	1	2	0.4 (0.0–1.6)	0.2 (0.0–0.8)
**Pacemaker**	10	3	4.0 (1.6–6.3)	0.4 (0.2–1.1)
**Supraventricular Tachycardia**	1	2	0.4 (0.0–1.6)	0.0
**Frequent supraventricular or ventricular premature beats**	31	54	14.0 (9.4–18.4)	7.4 (5.0–10.1)
**Major QT prolongation (>115%)**	13	13	5.3 (2.8–8.1)	4.0 (2.2–6.0)
**Left ventricular hypertrophy**	2	15	0.8 (0.4–2.0)	2.7 (1.3–4.2)

In the analysis of semi-competitive risks, the mean follow-up of the patients of the cohort was 89.6 months. The mean follow-up time until death was 92.9 months for the ChD group and 100.1 months for the NChD group. During this mean follow-up time, 108 patients in the ChD group and 237 patients in the NChD group died. The mean follow-up time until the occurrence of ECG abnormalities was 12.6 months for the ChD group and 20.4 months for the NChD group. In the multivariate analysis (Model 3), ChD is an independent risk of the occurrence of new major ECG abnormalities with an HR: 2.89 (95% CI 2.28–3.67) and is a risk for death with HR: 1.71 (95% CI 1.18–2.48) (Table 5). When the ChD and NChD groups present new or more ECG abnormalities, they have the same risk of death HR: 1.07 (95% CI 0.83–1.37).

**Table 5:** Influence of Chagas disease in comparison to the group without Chagas disease in the occurrence of new ECG abnormalities and in the mortality: the semi-competitive risk analysis.

	New ECG abnormalities HR (95% CI)	Death HR (95% CI)	Death after New ECG abnormalities occurrence HR (95% CI)
Chagas disease—model 1	3.86 (2.97–5.01)	3.06 (2.04–4.58)	1.17 (0.88–1.56)
Chagas disease—model 2	3.01 (2.35–3.87)	1.88 (1.25–2.83)	1.05 (0.82–1.36)
Chagas disease—model 3	2.89 (2.28–3.67)	1.71 (1.18–2.48)	1.07 (0.83–1.37)

Model 1: Univariate analysis; Model 2: Adjusted for age and gender; Model 3: Adjusted for age, gender, smoking, diabetes, creatinine, systolic pressure (each 10mmHg) and body mass index

**Fig 3:**
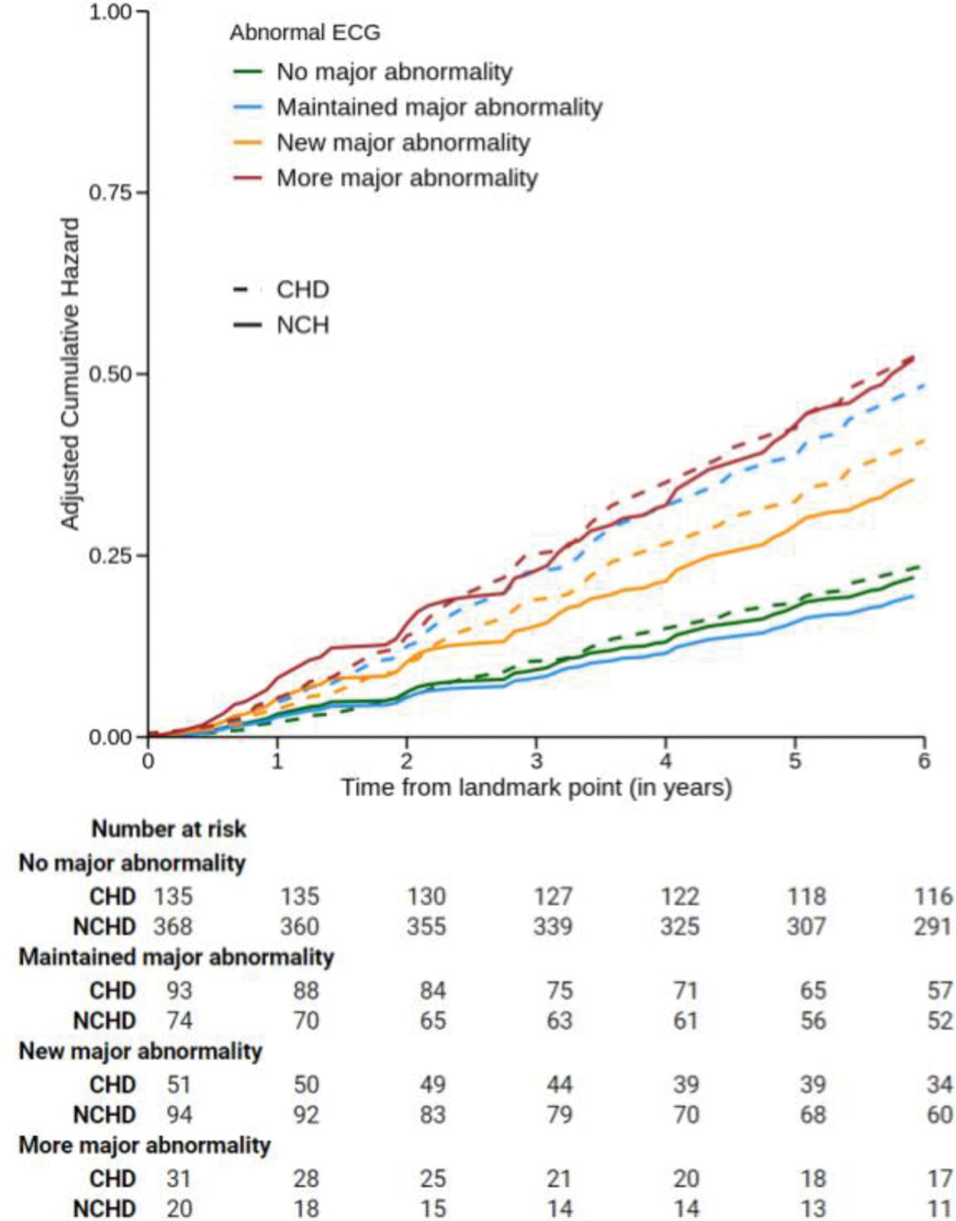
Cumulative incidence of death according to the incidence of ECG major abnormalities in the period comprising 1997–2002 in the group with Chagas disease and in the group without Chagas disease: a landmark analysis. The group Chagas disease (CHD) is represented by dotted lines: the subgroups Maintained (blue dotted line), New major (yellow dotted line) and More (red dotted line) major are significantly different from the group No major (green dotted line). The group Noninfected (NCH) is represented by continued lines: the subgroups New major (yellow continuous line) and More major (red continuous line) are significantly different from the subgroups Maintained (blue continuous line) and No major (green continuous line).

The landmark analysis (Fig 3) evaluated 866 individuals distributed as follows: Normal (ChD: 135 and NChD: 368), Maintained (ChD: 93 and NChD: 74), New (ChD: 51 and NChD: 94), and More (ChD: 31 and NChD: 20). In the individuals with ChD, compared with the group Normal (Table 6), the group Maintained had HR: 2.39 (95% CI 1.41–4.05), the group New had HR: 1.93 (95% CI 1.02–3.65), and the group More had HR: 2.69 (95% CI 1.38–5.24). Compared with the group Maintained, the group New had HR: 0.80 (95% CI 0.45–1.43), and the group More had HR: 1.12 (95% CI 0.61–2.05). In other words, having a second ECG with a major abnormality is uniformly related to a higher risk of death, independently of the first ECG.

**Table 6:** Risk of death according to the incidence of ECG major abnormalities in the period comprising 1997–2002 in the group with Chagas disease and in the group without Chagas disease using the group Normal as the reference: the landmark analysis.

ECG	Fully Adjusted Model * HR (95% CI)	P Value
**Chagas disease**		
Maintained	2.39 (1.41–4.05)	0.001
New	1.93 (1.02–3.65)	0.04
More	2.69 (1.38–5.24)	0.004
**Without Chagas disease**		
Maintained	0.87 (0.51–1.48)	0.62
New	1.69 (1.13–2.55)	0.01
More	2.62 (1.30–5.30)	0.007

* Adjusted for age, gender, smoking, diabetes, serum creatinine, systolic pressure (each 10mmHg), body mass index. CI, confidence interval

## Discussion

This population-based cohort study with a long-term follow-up of elderly individuals filled an important gap in the natural history of ChD in the old age. Even at advanced ages, independently of the time of the disease, individuals with ChD have a higher risk than non-infected persons of developing new major abnormalities in the ECG. A continuous excess in the incidence of major ECG abnormalities attributed to ChD was observed during the follow-up period independently of other covariates related to aging that could influence their evolution. The presence of major ECG abnormalities in a 2^nd^ ECG in the ChD group increases the risk of death independently of the individual maintained the abnormalities or developed new abnormalities. This unique study demonstrated that the chronic myocarditis expressed by the occurrence of new electrocardiographic abnormalities is in continuous progression.

This study advances our knowledge of ChD in the elderly over previous observations from this cohort, which showed an increased risk for mortality associated with *T*. *cruzi* infection in those aged 60 years old or more [16]. In a further analysis of the same cohort, we showed that major ECG abnormalities were more frequent in ChD patients, and the presence of any major ECG abnormalities doubled the risk of death in ChD elderly patients [9]. This study [9] identified a linear association between the prevalence of ECG abnormalities and age. However, no inference on the rate of development of cardiopathy in ChD and NChD subjects could be obtained from this cross-sectional data since repeated ECG had not been analyzed.

The present analysis results are compelling evidence that the *T*. *cruzi* infection is an independent risk for developing new ECG abnormalities in this community-based cohort of indwelling elderly, even when adjusted by co-variables and using the semi-competitive risk assessment for death as an alternative outcome. The incidence of new major ECG abnormality, when there is no major abnormality at the baseline, is also higher in ChD subjects. These results are similar to what was observed in the recently published NIH REDS cohort [17]; in ChD and NChD, blood donors have been followed from 2008–2010 to 2018–2019. The NIH REDS was comprised of Brazilian blood bank donors with ChD and NChD that were much younger (median age of 48 years, [Q1-Q3 40–57]) than those from the present study Bambui (68 [64–74]). In the NIH REDS cohort, individuals with ChD without cardiomyopathy, when compared with NChD group, had a HR: 2.24 (95% CI: 1.33–3.77) of developing left ventricular systolic dysfunction or enlarging the QRS complex ≥ 120ms. We found a similar risk of developing major ECG abnormality, which defines the presence of chronic cardiomyopathy in ChD, in the Bambui cohort (HR: 2.89 [95% CI 2.28–3.67]), even considering that the outcome is not identical. The Bambui cohort had a higher incidence of cardiomyopathy in the overall period of follow-up (24.8/ 1000 person-years) compared to the NIH REDS (13.8/ 1000 person-years). The incidence was higher probably because this cohort comprised older individuals, and all major ECG abnormalities were considered to characterize cardiomyopathy. The absolute incidence difference associated with ChD in the group with normal ECG in the baseline was 9.25 (95% CI 3.2–15.2) events/1000 py in the Bambui cohort of indwelling elderly, almost the same absolute incidence difference observed in the NIH REDS cohort, of predominantly of middle-aged adults (9.2 [95% CI, 3.6–15.0] events/1000 py).

Individuals in the ChD group that have Any major ECG abnormality in the baseline at 1997 have the same risk of developing new abnormalities as the NChD group that also already have Any major ECG abnormality in the baseline (Table 3). However, previous studies described that heart failure due to ChD has a worse prognosis than those due to other causes [18,19,20]. All of these studies are hospital-based cohorts of advanced cases, different to the Bambui cohort, which is a population-based study of indwelling subjects.

The ECG abnormalities with the highest incidence in this study were the RBBB and RBBB + AFB. Those abnormalities were related to death [9,21] and the progression to heart failure [21]. The ChD group also had an important incidence of premature beats and major Q waves abnormalities related to death in other cohorts [22,23,24]. Although low voltage is an established ECG abnormality related to the mortality in the general population with ChD [1], it was not included in the analyzes because it is not a major electrocardiographic abnormality of the Minnesota code. Furthermore, a previous study of this cohort suggested that it does not have prognostic value in the elderly individuals with ChD [9]. The findings of this study suggest that the *T*. *cruzi* infection provides continuous damage to the myocardium and that it is an independent risk of the occurrence of new ECG abnormalities despite the other factors that could cause cardiac lesions and promote ECG abnormalities associated with aging. The older age of these patients did not change the occurrence of new ECG abnormalities.

Moreover, the landmark analysis demonstrated that a ChD patient that develops a new major ECG abnormality in the follow-up visit has an increased risk of death, not different from the risk of those that already presented a major ECG abnormality in the baseline ECG. Thus, the newly developed cardiomyopathy in the elderly is as ominous as the cardiomyopathy established at earlier ages. This brand-new information can change the approach for the elderly ChD patient. Indeed, some current guidelines [1,25] recommend that specific anti-trypanosome drugs should be given preferentially to ChD patients under 50 years old. Our data stresses that avoidance of the development of cardiomyopathy is still important in old age and that specific treatment of elderly ChD patients should be considered more emphatically.

A recent systematic review of studies that evaluated the incidence of progression to ChCM in patients with the indeterminate ChD found 23 studies from 1960 and 2005: the ages ranged from 10 to 44 years, with a mean age of 31 years [4]. The lack of older people in these studies and the small population analyzed led to the mistaken conclusion that this group would have a more benign course of the disease [26,27]. One of the most important studies that evaluated the use of Benznidazole excluded individuals over 50 years of age to avoid misinterpretations of electrocardiographic changes [28]. These facts associated with the significant incidence of adverse effects of Benznidazole [29] led the guidelines and researchers not to value its prescription in the elderly [1,25,30]. However, there is growing evidence that treating individuals in the indeterminate form can reduce disease progression and mortality [28,31,32,33]. The results of this study indicate that the elderly in the indeterminate form of ChD could also benefit from treatment with Benznidazole. However, further studies are needed to clarify the effectiveness of the current anti-parasite drug regimens in this age range.

Although the study’s main goal was to evaluate the incidence of major ECG abnormalities in the elderly population with ChD, it also sheds light on the incidence in the non-infected elderly in Brazil. It was suggested that ECG abnormalities could improve risk estimation for cardiovascular disease in healthy asymptomatic elderly [34,35] and that each population has its own risk for their occurrence [36]. In this cohort, when the ChD and NChD groups developed ECG abnormalities, they had the same risk of death. When the risk of death was stratified in the Landmark analysis, the group NChD New and More had a higher risk HR: 1.69 (95% CI: 1.13–2.55) and HR: 2.62 (95% CI: 1.30–5.30).

The most incident abnormalities in the NChD group were frequent supraventricular or ventricular premature beats followed by Major isolated ST-T abnormalities and major Q—wave abnormalities. The first ones have been associated with reduced left ventricular ejection fraction, incident heart failure, and death in other studies [36,37,38]. Considering that symptoms of coronary heart disease may be missing or atypical in the elderly, the incidence of major ST-T abnormalities isolated or associated with major Q-waves abnormalities in NChD individuals is an important marker for myocardial infarction and death in clinical practice and epidemiological studies [39,40]. In the ELSA- Brasil cohort, Major isolated ST-T abnormalities also increased the risk of death [41]. Thus, the incidence of these abnormalities is a marker of worse evolution of the NChD group. The unadjusted incidence of atrial fibrillation in the NChD group was similar to other elderly populations [42,43,44]; it increases with advancing age and is associated with excess mortality [42]. Knowing these incidences is important for the planning of the health system.

### Limitations

The left ventricular ejection fraction, which is the main prognostic risk factor in most cardiomyopathies, was not measured in the individuals in this cohort. Holter data is also missing. This could identify ECG changes at baseline and follow-up, and non-sustained Ventricular Tachycardia as an abnormality that increases the risk of death could also be an adjustment factor to be included in multivariate models. It must be acknowledged that frequent supraventricular premature beats and left ventricular hypertrophy are not used regularly as typical ECG abnormalities for the diagnosis of ChD progression to the cardiac form. The inclusion of these abnormalities may have overestimated the progression rate to Chagas disease cardiomyopathy.

This research did not evaluate the polymerase-chain-reaction assay for *Trypanosoma cruzi*. It would suggest the persistence of the parasite, which may be an important factor in conjunction with individual host factors to trigger the inflammatory process [17,45]. There is no information about the use of Benznidazole for the treatment of the individuals in this cohort. The previous treatment could have prevented the evolution of cardiomyopathy in some individuals [31,32]. It should be considered that some of the patients with ChD who developed heart failure in the follow-up initiated treatment that prevented the development of new ECG abnormalities such as atrial fibrillation or premature beats or even led to their regression. Such treatment could also have reduced the mortality of the patients with ChD who developed heart failure or arrhythmias. We did not have access to the changes in the patient’s medication during the follow-up. Implantable cardioverter-defibrillator or pacemaker implant in the individuals of the cohort are another competing risk that the results were not adjusted for. A device implant could have postponed the death event.

Between 1997 and 2002, 19.4% of individuals in the ChD group and 14.2% of individuals in the NChD group died. In the same period, 13.4% of the individuals in the ChD group did not undergo the ECG, as well as 13.4% of those in the NChD group. Losses resulting from deaths occurred in greater proportions in the group with ChD and are related to individuals with more severe disease. Although this may have influenced the results, semi-competitive risk survival analysis was used to correct this impact. In addition, each survival analyzes were also adjusted for risk factors for cardiovascular disease. Losses resulting from the lack of ECG occurred randomly in both groups with similar proportions. This is an intrinsic limitation of cohort studies and was corrected by adjustments made in survival analyses.

Mortality was not analyzed by causes because it could reduce the power of the analysis. Several analyses were performed, including subgroups of patients related to the serological status, the presence of ECG abnormality on the baseline and the development of new ECG abnormalities. To categorize the cause of death would lead to a reduction of the number of outcomes by groups of exposition and would preclude the detailed analysis performed.

### Strengths

This is a large population-based cohort that represents the elderly patients with ChD living in the community followed for a long period of 14 years. Patients with ChD were compared to NChD individuals residing in the same community, and the analyses were adjusted to confounding factors. These are important points in the design of this study that contribute to the applicability of our findings to other populations. The electrocardiograms were analyzed by the Minnesota Code, an international standard that makes our analysis objective and reproducible in other studies and clinical settings. We also used more advanced statistical methods than previous research, that allowed us to have a broader picture of the ECG evolution.

### Conclusion

This is the first study to evaluate electrocardiographic evolution in a large elderly population with ChD repeating the ECG in the same individuals. It showed that even at advanced ages, persons with ChD have a higher risk than the non-infected of developing new abnormalities in the ECG. Individuals who develop cardiomyopathy have a higher risk of death. These alterations seem to be a genuine clinical expression that the disease continues to progress even in the elderly, in whom the ChCM is still a major cause of death.
